# Validation of the Swedish Dynamic Imaging Grade of Swallowing Toxicity for Flexible Endoscopic Evaluation of Swallowing (DIGEST-FEES)

**DOI:** 10.1007/s00455-024-10759-2

**Published:** 2024-09-27

**Authors:** Kerstin Petersson, Caterina Finizia, Nina Pauli, Lisa Tuomi

**Affiliations:** 1 Department of Otorhinolaryngology, Head and Neck Surgery, Institute of Clinical Sciences, Sahlgrenska Academy, University of Gothenburg, Gothenburg, Sweden; 2 Department of Otorhinolaryngology, Head and Neck Surgery, Sahlgrenska University Hospital, Region Västra Götaland, Gothenburg, SE-41345 Sweden; 3 Department of Otorhinolaryngology at Högsbo Specialist Hospital, Region Västra Götaland SV Hospital group, Gothenburg, Sweden; 4 Institute of Neuroscience and Physiology, Speech and Language Pathology Unit, Sahlgrenska Academy, University of Gothenburg, Gothenburg, Sweden

**Keywords:** Head and neck cancer, Flexible endoscopic evaluation of swallowing, Dysphagia

## Abstract

In the head and neck cancer (HNC) population around 45% suffer from chronic swallowing difficulties after cancer treatment. Previously a measure for flexible endoscopic evaluation of swallowing (FEES) where swallowing efficiency, safety and overall ability is evaluated within the same framework has been lacking. The Dynamic Imaging Grade of Swallowing Toxicity for FEES (DIGEST-FEES) was developed in 2021 and provides such a measure for patients with HNC. The aim of this study was to translate and validate the DIGEST-FEES into Swedish (Sw-DIGEST-FEES). A translation of the protocol to Swedish was done through forward-backward translation. Two raters rated eighty-nine FEES recordings according to the Sw-DIGEST-FEES and five reference measures of swallowing ability: Yale Pharyngeal Residue Severity Rating Scale, Swallowing Performance Scale, Murray Secretion Scale, MD Anderson Dysphagia Inventory and Penetration Aspiration Scale. Intra- and interrater reliability was analyzed. Construct validity was evaluated by correlating the Sw-DIGEST-FEES ratings to the reference measures. A priori hypothesis was that the correlations would correspond to those of the reference measures included in the original English version. The Sw-DIGEST-FEES demonstrated retained psychometric properties. Construct validity was good. 79% of correlations to the reference measures were equal to or stronger than those in the original development. Inter-rater agreement of the Sw-DIGEST-FEES ranged from substantial to almost perfect (0.76–0.81). Intra-rater reliability was in general almost perfect (0.8-1). The Sw-DIGEST-FEES can be considered a valid and reliable protocol for use in evaluation of swallowing function in HNC patients.

## Introduction

The prevalence of swallowing difficulties (dysphagia) is high among patients with head and neck cancer (HNC), with around 45% suffering from chronic swallowing difficulties after cancer treatment [1]. There are several causes of swallowing difficulties in HNC patients. Impairment can arise as a complication due to the growth of the tumor or as an adverse event after radiotherapy or surgical treatment. Both surgical treatment and radiotherapy can cause restrictions in swallowing physiology and altered oral and pharyngeal sensitivity [2]. Swallowing difficulties may result in medical complications such as malnutrition, dehydration, aspiration pneumonia, and lower health-related quality of life (HRQL) [2]. Researchers and clinicians struggle to find effective ways to prevent and rehabilitate swallowing difficulties in this patient population, and the diverging measures used to evaluate swallowing interventions complicate these efforts.

Flexible endoscopic evaluation of swallowing (FEES) is a well-established instrumental assessment of swallowing function and can be used in most patient populations, including HNC. Swallowing function is usually determined by an assessment of swallowing safety, i.e. severity of aspiration or penetration events, and swallowing efficiency, i.e. level of pharyngeal residue after bolus clearance. Other variables, such as salivary secretions and swallowing physiology, i.e. movements in structures during swallowing, may also be included [3]. While these aspects of swallowing function can be determined by a variety of available outcome measures, there is no ‘gold standard’ in research or clinical practice. Consequently, it is difficult to evaluate and compare results across research studies [4–6]. Furthermore, the validity of most measures is under debate as their psychometric properties lack robust assessment [3].

The penetration-aspiration scale (PAS) is widely used in research studies where swallowing safety is evaluated by instrumental assessment, but it has some disadvantages [7, 8]. PAS is an ordinal scale describing how far into the airway the bolus passes. However, higher PAS scores do not always reflect a more severely impaired swallowing ability as this also depend on the amount of penetration/aspiration and on how well patients are able to protect the airway by coughing or clearing the throat [9]. Furthermore, there is no gold standard for reporting PAS results, even though a majority of studies uses the patients worst PAS score as an overall reflection of swallowing safety [7].

The Dynamic Imaging Grade of Swallowing Toxicity for FEES (DIGEST-FEES) provides an aggregated metric of swallowing function, which had previously been lacking for FEES. The scale was developed in 2021 for the HNC population and has sound psychometric properties [10]. The scale is an adaptation of the DIGEST method originally developed for videofluoroscopic assessment of swallowing function (VFS). A 2019 review of swallowing measures for VFS and FEES deemed that DIGEST had superior psychometric properties to most other measures within the field [3]. The DIGEST-FEES rests on the same methodology. Both methods consist of three scales: safety, efficiency, and an overall grade. In the DIGEST-FEES, swallowing safety (per the penetration-aspiration scale [PAS]) is rated for each bolus, however, the rating also depends on the frequency of the worst PAS, the volume of penetration/aspiration, and the bolus consistency. This approach measures the severity of airway invasion with improved ordinal qualities compared to the PAS [9]. The DIGEST-FEES efficiency scale rates the residue left in the pharynx based on the maximum amount of residue and the consistency of the bolus. The safety and efficiency ratings are then synthesized to obtain the overall DIGEST-FEES grade, representing overall swallowing function. The DIGEST-FEES scale is compatible with the Common Terminology Criteria for Adverse Events (CTCAE) frequently used to assess the severity of adverse events after cancer treatment and aids the communication of swallowing impairment within the oncology field [11]. The widespread use of DIGEST-FEES could permit the international standardization of swallowing assessments both in research and clinical settings.

To date, there is no equivalent comprehensive validated swallowing measure for FEES, and the DIGEST-FEES scale is not yet available in Swedish. The present study aimed to translate and validate the DIGEST-FEES for the Swedish HNC population by using five well-known dysphagia instruments as psychometric anchors.

## Method

### Participants and Video Recordings

The FEES recordings included in the study were originally collected in a randomized controlled trial in which participants either performed the head-lift exercise for 8 weeks or were managed according to routines at the local hospital [12]. The inclusion criteria were patients with HNC who completed radiotherapy 6–36 months prior to recruitment and had swallowing difficulties according to PAS ≥ 2 (established by VFS). FEES was performed at baseline and after 8 weeks of intervention. Eighty-nine FEES recordings from 47 participants were used in the validation. Forty-two participants had two FEES recordings, and five had one recording each.

The bolus protocol during FEES is described according to the International Dysphagia Diet Standardization Initiative (IDDSI) [13]. Boluses were distributed as follows: 3 ml, 5 ml, and 10 ml of mildly thick liquid (IDDSI 2); 3 ml, 10 ml, and 20 ml (sequential drinking) of thin liquid (IDDSI 0); and one-fourth of a soft biscuit (IDDSI 7). Liquids were colored with green caramel coloring.

All ratings of swallowing function were rated by two speech and language pathologists (SLPs) with more than five years of experience in performing and assessing FEES. Joint training sessions in the assessment protocols were held prior to rating the recordings. The analysis of recordings was blinded and each rater performed the analysis separately.

### Translating the Dynamic Imaging Grade of Swallowing Toxicity for FEES (DIGEST-FEES)

The translation of DIGEST-FEES was conducted using a forward-backward translation method [14]. The original English version of DIGEST-FEES was translated into Swedish by two experienced clinicians and researchers within the field of swallowing disorders; both were native Swedish speakers with a good knowledge of the English language. The two independent Swedish translations were synthesized by two clinicians with over 10 years of experience in swallowing disorders. The consensus version was then translated back to English by a bilingual physician with extensive knowledge within the field of HNC but was not familiar with the DIGEST-FEES. Discrepancies between the original version and the English back-translation were discussed with the original developers and the Swedish version of the scale, hereafter Sw-DIGEST-FEES, was then finalized.

### Comparison of the Swedish Translation to Reference Measures

The FEES recordings of HNC patients were rated according to the Sw-DIGEST-FEES. To determine construct validity, the ratings were compared to reference measures that were either previously validated or widely used and also corresponded to measures used in the validation of the original version of the DIGEST-FEES [10]. The following reference measures were used:

1. The PAS scale measures airway protection; it is a widely used and reliable scale with ratings that range from 1 to 8 (normal to silent aspiration) [8].
2. The Yale pharyngeal residue severity rating scale is considered a valid and reliable measure of residue in the vallecula and pyriform sinuses and is graded according to a 5-point scale (no residue to severe residue) [15, 16].
3. The Murray secretion scale measures pharyngeal secretions, with scores ranging from 1 to 4 (no visible secretions/transient bubbles to secretions in the laryngeal vestibule). The scale is valid and reliable and has been shown to predict aspiration [17–19].
4. The Swallowing Performance Scale (SPS) has been used to measure overall swallowing ability in HNC cohorts in previous research studies and was found to correlate well with physician-rated swallowing ability. The scale rates the degree of impairment from normal (1) to severe (7) [20, 21].
5. The MD Anderson Dysphagia Inventory (MDADI) measures subjective swallowing function and dysphagia-specific HRQL and was developed specifically for patients with HNC [22]. The MDADI consists of 20 items within four domains: emotional, functional, physical, and global. Responses range from strongly agree to strongly disagree, rated on a 5-point Likert scale. Total scores range from 20 to 100, where a higher score represents better functioning. The questionnaire is widely used, valid, and reliable.

### Statistical Analysis

Descriptive statistics was used to report participant characteristics. Categorical variables were described by numbers and percentages, and continuous variables by the mean, standard deviation (SD), minimum, and maximum.

For assessment of construct validity, Spearman correlation coefficients (*p* ≤ 0.05) were calculated for the Sw-DIGEST-FEES safety and efficiency ratings and overall grade for each reference measure, i.e. MDADI total, MDADI emotional, MDADI functional, MDADI physical, MDADI global, SPS, Murray secretion scale, Yale pharyngeal residue severity rating scale (vallecula and pyriform sinus), and PAS. The a priori hypothesis was that the included reference measures in the Swedish translation would perform similarly to those used in the validation of the original version of DIGEST-FEES, i.e. moderate agreement for reference measures of overall swallowing ability and secretions, fair for the MDADI, and substantial to almost perfect for residue ratings [10].

Inter-rater reliability was based on Sw-DIGEST-FEES ratings for all 89 FEES recordings. Nineteen recordings were doubled (21%) to evaluate the intra-rater reliability of the translated scale for both raters. Inter- and intra-rater reliability were assessed using weighted kappa (κw) and percentage of exact agreement for the safety, efficiency, and overall Sw-DIGEST-FEES grade. Correlations for construct validity and weighted kappa were interpreted according to the following ranges: ≤0, less than chance agreement; 0.0-0.20, slight agreement; 0.21–0.40, fair agreement; 0.41–0.60, moderate agreement; 0.61–0.80, substantial agreement; and 0.81-1.00, almost perfect agreement [23].

## Results

**Fig. 1:**
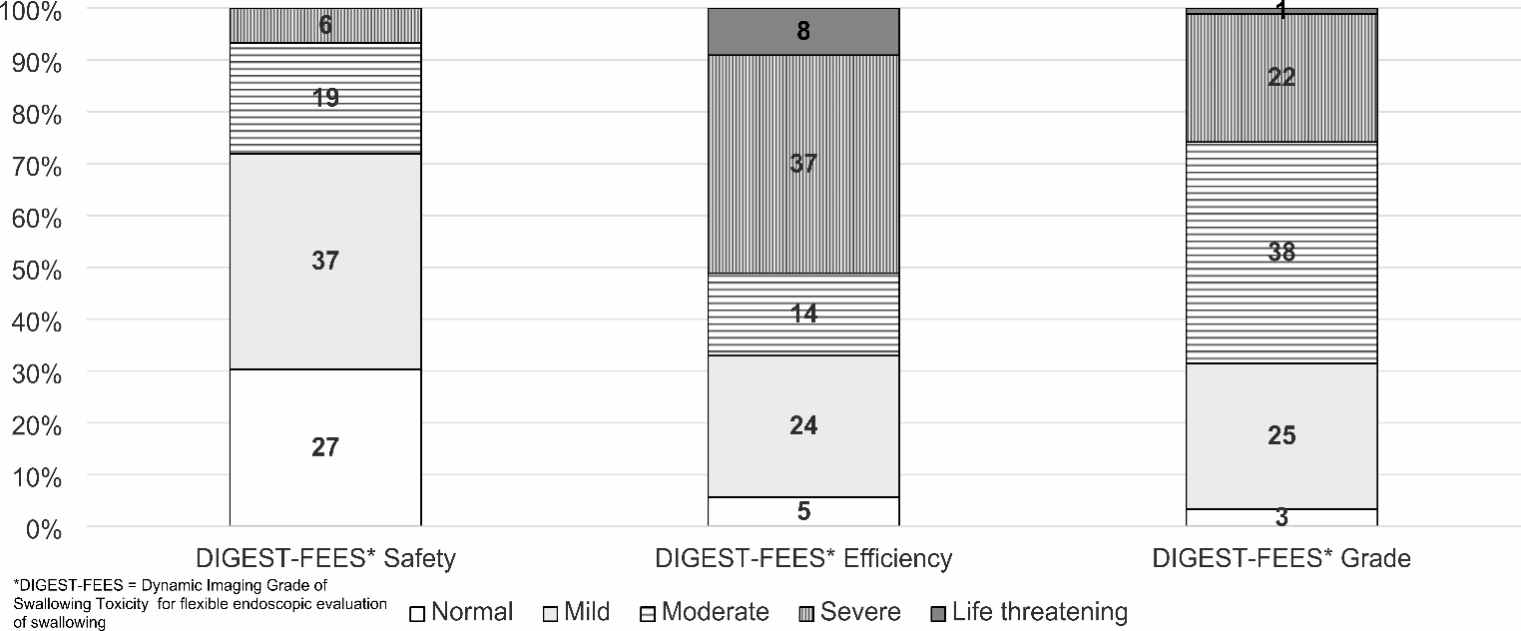
Ratings from the Swedish DIGEST-FEES* expressed in frequency and percent (*n* = 89)

Participant characteristics are presented in Table 1. Most participants were male (77%) and treated for a tumor in the tonsil (43%) or the base of the tongue (38%). The majority of participants (87%) received treatment with modern radiotherapy techniques, intensity modulated/volumetric modulated radiation therapy (IMRT/ VMAT), while 13% had conventional therapy, i.e., 3D-CRT. The reference measures revealed swallowing impairment ranging from mild to severe based on the participants’ FEES recordings (Table 2). All PAS scores were represented. Yale pharyngeal residue severity rating scale scores ranged from trace to severe for both vallecula and piriform sinus residue. The mean MDADI total score, reflecting patients’ subjective perception of their swallowing function, was 72.8 points (SD = 17). The distribution of ratings on the Sw-DIGEST-FEES scales is presented in Fig. 1. The participants’ efficiency scale scores and the overall DIGEST-FEES grades ranged from normal to life-threatening. The safety scale had a larger proportion of normal ratings than the other two scales and no life-threatening ratings.

**Table 1:** Tumor and treatment characteristics of the study participants

Variable	*n* = 47‡
	Mean (SD) (min; max)
**Age**	63.1 (7.4) (45;80)
**Time since end of radiotherapy (months)**	12.2 (7.2) (6;37)
	**n (%)†**
**Sex**	
Male	36 (77)
Female	11 (23)
**Tumor localization**	
Tonsil	20 (43)
Base of tongue	18 (38)
Larynx	7 (15)
Hypopharynx	2 (4)
**Tumor stage**	
Early (I-II)	9 (19)
Advanced (III-IV)	38 (81)
Comorbidity (ACE 27*)	
None	21 (45)
Mild	21 (45)
Moderate	5 (11)
**Radiotherapy**	47 (100)
**Chemotherapy**	37 (79)
**Brachytherapy**	15 (32)
Number of FEES* recordings	
Participation in one FEES*	5 (11)
Participation in two FEES*	42 (89)

* *Abbreviations* ACE-27 = Adult Comorbidity Evaluation-27, FEES = Flexible endoscopic evaluation of swallowing

†Percentages rounded, therefore does not always sum to 100

**Table 2:** Descriptive data on swallowing function according to reference measures

Measurement of swallowing ability			Mean (SD) (min; max)
MDADI* -total score (*n* = 47)			72.8 (17) (35;95)
			FEES* recordings *n* = 89
			n (%)
Swallowing Performance Scale (SPS)			
1	Normal	4 (5)	
2	Functional	8 (9)	
3	Mild impairment	26 (29)	
4	Mild-moderate impairment	23 (26)	
5	Moderate impairment	13 (15)	
6	Moderate-severe impairment	15 (17)	
7	Severe impairment	0 (0)	
Penetration Aspiration Scale (PAS)			
1	Normal	12 (14)	
2	Transient penetration	3 (3)	
3	Penetration above vocal folds	22 (25)	
4	Transient penetration vocal folds	5 (6)	
5	Penetration on vocal folds	27 (30)	
6	Transient aspiration	3 (3)	
7	Aspiration despite clearing effort	7 (8)	
8	Silent aspiration	10 (11)	
Murray secretion scale			
1	Normal	34 (39)	
2	Mild	27 (31)	
3	Moderate	4 (5)	
4	Severe	22 (25)	
	Missing	2	
Yale pharyngeal residue severity rating scale - vallecula			
1	None	0 (0)	
2	Trace	10 (11)	
3	Mild	28 (32)	
4	Moderate	27 (30)	
5	Severe	24 (27)	
Yale pharyngeal residue severity rating scale – pyriform sinuses			
1	None	0 (0)	
2	Trace	27 (30)	
3	Mild	42 (47)	
4	Moderate	16 (18)	
5	Severe	4 (5)	

* *Abbreviations* FEES = Flexible endoscopic evaluation of swallowing, MDADI = the MD Anderson Dysphagia Inventory- scores range from 20–100 and a higher score represents better function

### Construct Validity

Correlations between the reference measures and the Sw-DIGEST-FEES safety and efficiency scores and overall grade varied from fair to substantial (Table 3). The correlations between instrumental reference measures and the Sw-DIGEST-FEES varied from fair to substantial. In general, patient-reported swallowing ability correlated moderately to the Sw-DIGEST-FEES ratings.

**Table 3:** Comparison between the Swedish translation of the dynamic imaging Grade of swallowing toxicity for flexible endoscopic evaluation of swallowing (Sw-DIGEST-FEES) and the reference measures

Measure	Sw-DIGEST-FEES* safety	Sw-DIGEST-FEES* efficiency	Sw-DIGEST-FEES* grade
	Correlation coefficient p-value	Correlation coefficient p-value	Correlation coefficient p-value
**Penetration Aspiration Scale (Worst PAS)**	0.48 < 0.0001	0.35 0.0007	0.67 < 0.0001
**Yale pharyngeal residue severity rating scale (vallecula)**	0.72 < 0.0001	0.78 < 0.0001	0.62 < 0.0001
**Yale pharyngeal residue severity rating scale (pyriform sinuses)**	0.58 < 0.0001	0.48 < 0.0001	0.62 < 0.0001
**Swallowing performance scale (SPS)**	0.70 < 0.0001	0.61 < 0.0001	0.80 < 0.0001
**Murray secretion scale**	0.40 < 0.0001	0.39 0.0003	0.41 < 0.0001
MDADI* total	-0.44 < 0.0001	-0.48 < 0.0001	-0.42 < 0.0001
MDADI* emotional	-0.42 < 0.0001	-0.44 < 0.0001	-0.38 0.0002
MDADI* functional	-0.43 < 0.0001	-0.49 < 0.0001	-0.38 0.0002
MDADI* physical	-0.46 < 0.0001	-0.49 < 0.0001	-0.45 < 0.0001
MDADI* global	-0.29 0.0065	-0.31 0.0034	-0.23 0.0289

* *Abbreviations* Sw-DIGEST-FEES = the Swedish translation of Dynamic Imaging Grade of Swallowing Toxicity for flexible endoscopic evaluation of swallowing, MDADI = the MD Anderson Dysphagia Inventory

In MDADI scores, a higher score indicates better swallowing function, in all other measures, a higher score indicates worse function

Correlations were interpreted per Cohen’s standard as: ≤0 = less than chance agreement, 0.0-0.20 = slight agreement, 0.21–0.40 = fair agreement, 0.41–0.60 = moderate agreement, 0.61–0.80 = substantial agreement and 0.81-1.00 almost perfect

### Reliability Analysis

Inter-rater agreement was substantial for the Sw-DIGEST-FEES safety and overall grade and almost perfect for efficiency. Rater two had an almost perfect intra-rater reliability on all three scales, while rater one had an almost perfect agreement for safety and overall grade and substantial agreement for efficiency.

## Discussion

In this study, we translated and validated the DIGEST-FEES for the Swedish HNC population. Based on correlations to five well-known dysphagia instruments as psychometric anchors, the Sw-DIGEST-FEES can be considered a valid and reliable scale to evaluate swallowing function in Swedish HNC patients.

In defining good construct validity, it has been suggested that 75% of results should be in accordance with the a priori hypotheses [24]. In the present study, 19 of 24 correlations (79%) to included reference measures were equal to, or better than those in the validation of the original English version of the DIGEST-FEES.

### Sw-DIGEST-FEES Safety

The correlations between the Sw-DIGEST-FEES safety rating and instrumental reference measures were mostly moderate to substantial and were similar to those reported in the validation of the DIGEST-FEES. Agreement was stronger (substantial) between the safety rating and measures of overall swallowing ability in the present study using SPS. In contrast, the original development and validation study used the Functional Oral Intake Scale (FOIS), and the correlation was fair [10, 25]. This likely reflects differences in the rating procedure across the two studies. The SPS was rated by the SLPs based on the FEES recordings while FOIS ratings were based on patient reports on what consistencies of food they included in their diet [10].

Moreover, we included PAS as a reference scale in the validation of the Sw-DIGEST-FEES, but it was not included in the development study of the DIGEST-FEES [10]. In the present study, the correlation was stronger to the safety rating and the Sw-DIGEST-FEES grade (moderate and substantial) than to the efficiency rating (fair). These results illustrate the differences between the PAS and the safety scale. Although 11% of the FEES recordings contained events of silent aspiration, corresponding to the highest PAS score, none were rated as life-threatening on the DIGEST-FEES safetyscale as the frequency and the aspirated amount had also been considered. This shows that only assessing swallowing ability based on the worst PAS score can risk overstating the severity of the impairment and highlights the unique qualities of the safety rating. It also explains why the two scales, which measure the same ability, had only a moderate agreement.

### Sw-DIGEST-FEES Efficiency

The correlation between the Yale pharyngeal residue severity rating scale and the efficiency rating was stronger in the original validation for both vallecular and pyriform sinus residue than for Sw-DIGEST-FEES [10]. In DIGEST, residue is rated after the first bolus swallow, however, in the present study, the Yale pharyngeal residue severity rating was made after the first clearing swallow. The agreement between the Yale pharyngeal residue severity rating scale and the Sw-DIGEST-FEES efficiency rating would probably have been stronger if both ratings had been carried out after the first swallow.

In terms of secretion severity, the correlation between secretion measures and the efficiency rating was weaker for the Sw-DIGEST FEES (fair) than in the original development and validation study (moderate) [10], which could be explained in part by the use of different measures Murray secretion scale in the current study and the Secretion Severity Scale in the original study. Regardless, a fair to moderate correlation between secretions and swallowing efficiency seems reasonable. The presence of mild secretions is indicative of a more impaired swallowing efficiency than mild residue. For example < 10% residue is considered normal in the DIGEST-FEES methodology [10], but would result in at least a mild secretion rating on the Murray secretion scale.

### Sw-DIGEST-FEES Grade

Correlations between all reference measures and the Sw-DIGEST-FEES grade were comparable to those in the validation of the original DIGEST-FEES, i.e., substantial to moderate except for overall swallowing ability, which was stronger in the present study (SPS vs. FOIS). This indicates that the Sw-DIGEST-FEES grade demonstrates sufficient validity and reliability.

### Self-reported Dysphagia-specific Health-related Quality of Life

The correlations between the Sw-DIGEST-FEES ratings and all MDADI domains were in general weaker than instrumental measures of swallowing function, both in the present study and in the original development [10]. However, this is not surprising, since several studies have shown that the correlation between instrumental evaluation of swallowing ability and self-reported dysphagia-specific HRQL is generally weak [26–28].

### Inter- and Intra-rater Reliability

The Sw-DIGEST-FEES ratings demonstrated sufficient reliability. Inter-rater reliability agreement was substantial for the Sw-DIGEST-FEES grade (0.77), while intra-rater reliability was almost perfect for both raters. A weighted kappa ≥ 0.7 is suggested as a cut-off for good reliability [24]. In the development and validation of the DIGEST-FEES, inter-rater reliability for the overall grade was even higher and reached an almost perfect agreement (0.83) [10].

### Limitations and Strengths

The present study has several limitations. We did not investigate the sensitivity and responsiveness of the Sw-DIGEST-FEES in the target population. However, the ordinality of the scale has been previously established for DIGEST for VFS using the same methodology [29]. In addition, content validity was not examined as this was done thoroughly in clinical conditions applicable to a Swedish HNC population – both in the development of DIGEST and in its adaptation to FEES [10, 11, 29]. A further limitation is that all participants had been treated with radiotherapy and there was no representation of surgically treated HNC patients.

The present study has several strengths. The translation process followed recommended procedures through a forward-backward translation method in collaboration with the developers of the original English version of the DIGEST-FEES. The validity and reliability of the Sw-DIGEST-FEES were tested on a fairly large sample of 89 FEES recordings and correlated with five reference measures. Important to the validation of the measure, the study population had all levels of swallowing ability represented in the Sw-DIGEST-FEES grade, even though the proportion of mild, moderate, and severe impairment was larger compared to normal and life-threatening levels.

## Conclusion

The present study provides clinicians and researchers with a valid and reliable Swedish version of the DIGEST-FEES, which will help improve swallowing assessments and facilitate the comparison of research findings and clinical outcomes – both in a Swedish context and internationally. It also gives access to a valuable tool that communicates the level of swallowing impairment and is compatible with the well-known CTCAE terminology used by healthcare practitioners within oncology.

## Data Availability

Data cannot be shared publicly because the ethical approval for this study applies for reports of data on a group level, not an individual level. Therefore, data for individual cannot be made publicly available.
